# Porcine epidemic diarrhea virus nsp14 inhibits NF-κB pathway activation by targeting the IKK complex and p65

**DOI:** 10.1186/s44149-021-00025-5

**Published:** 2021-10-14

**Authors:** Shasha Li, Fan Yang, Caina Ma, Weijun Cao, Jinping Yang, Zhenxiang Zhao, Hong Tian, Zixiang Zhu, Haixue Zheng

**Affiliations:** State Key Laboratory of Veterinary Etiological Biology, National Foot and Mouth Diseases Reference Laboratory, Key Laboratory of Animal Virology of Ministry of Agriculture, Lanzhou Veterinary Research Institute, Chinese Academy of Agricultural Sciences, Lanzhou, Gansu 730046 China

**Keywords:** CoVs, PEDV, nsp14, NF-κB, innate immunity

## Abstract

Coronaviruses (CoVs) are a group of related enveloped RNA viruses that have severe consequences in a wide variety of animals by causing respiratory, enteric or systemic diseases. Porcine epidemic diarrhea virus (PEDV) is an economically important CoV distributed worldwide that causes diarrhea in pigs. nsp14 is a nonstructural protein of PEDV that is involved in regulation of innate immunity and viral replication. However, the function and mechanism by which nsp14 modulates and manipulates host immune responses remain largely unknown. Here, we report that PEDV nsp14 is an NF-κB pathway antagonist. Overexpression PEDV nsp14 protein remarkably decreases SeV-, poly (I:C)- and TNF-α-induced NF-κB activation. Meanwhile, expression of proinflammatory cytokines is suppressed by nsp14. nsp14 inhibits the phosphorylation of IKKs by interacting with IKKs and p65. Furthermore, nsp14 suppresses TNF-α-induced phosphorylation and nuclear import of p65. Overexpression nsp14 considerably increases PEDV replication. These results suggest a novel mechanism employed by PEDV to suppress the host antiviral response, providing insights that can guide the development of antivirals against CoVs.

## Introduction

Coronaviruses (CoVs) are a group of related enveloped RNA viruses within the family of *Coronaviridae* in the order of *Nidovirales* that consist of four genera, *Alphacoronavirus*, *Betacoronavirus*, *Deltacoronavirus* and *Gammacoronavirus* (Gorbalenya et al. 2004; Woo et al. 2012). CoVs have severe health consequences by causing respiratory, enteric or systemic diseases in various animals. Some CoVs are lethal to their hosts, such as the CoVs that cause severe acute respiratory syndrome (SARS), Middle-east respiratory syndrome (MERS) and COVID-19 in humans. Certain CoVs, including infectious bronchitis virus (IBV), porcine epidemic diarrhea virus (PEDV), and ferret systemic coronavirus (FSC), are lethal to animals (Haake et al. 2020).

CoVs have positive single-stranded RNA viral genomes ranging from 25 to 32 kb, which encode a series of structural, accessory and nonstructural proteins. Structural proteins conclude nucleocapsid (N), membrane (M), spike (S), and envelope (E) proteins (de Artika et al. 2020), and ORF3 encodes a hypothetical accessory protein. Two large open reading frames (ORFs), ORF1a and ORF1b, compose of major part of the viral genome and encode two large replicase polyproteins (pp1a and pp1ab), which are subsequently cleaved by viral proteases into 16 nonstructural proteins (nsps) (Ziebuhr et al. 2000). These nsps, together with other viral proteins and cellular factors, assemble into a large replication-transcription complex (RTC). RTCs are associated with double membrane vesicles derived from the endoplasmic reticulum and are responsible for viral RNA replication and transcription of subgenomic RNAs.

The innate immune response is critical for defending the host from various invading pathogens. Viral pathogen-associated molecular patterns (PAMPs) are recognized by pattern recognition receptors (PRRs), which induce the production of inflammatory cytokines and type I interferons (IFNs) by activating transcription factor nuclear factor kappa B (NF-κB) and IFN regulatory factors. Activation of NF-κB signaling pathway is crucial for innate immunity and other processes involving cellular survival, proliferation and differentiation. NF-κB family consists of five members: p50, p52, p65, RelB and c-Rel (Hayden and Ghosh 2008). Classical NF-κB signaling pathway activation requires the release of NF-κB p50/p65 dimers, while nonclassical NF-κB signaling pathway activation requires the formation of p52/Rel B dimers. In classical NF-κB signaling pathway, p65/p50 dimers are sequestered in cytoplasm through interaction with an inhibitors of NF-κB (IκBα) (Rothwarf et al. 1998). Upon viral infection, IκBα is phosphorylated by IκB kinase (IKKα and IKKβ) complex and degraded in proteasome, thereby releasing p65/p50 dimers for phosphorylation and translocation into nucleus (Kanarek and Ben-Neriah 2012; Liu et al. 2012). The major upstream receptors mediating NF-κB activation include toll-like receptors (TLRs), retinoic acid-inducible gene I (RIG-I), tumor necrosis factor receptor (TNFR), and interleukin 1 receptor type 1 (IL-1R1). The downstream proteins regulated by these receptors mainly include myeloid differentiation primary response gene 88 (MyD88), Toll/IL-1 receptor (TIR)-containing adaptor-inducing IFN-β (TRIF), and mitochondrial antiviral signaling protein (MAVS).

To establish successful infection, various CoVs have evolved multiple strategies to evade the host antiviral response. During CoV infection, several replicase proteins functioned as interferon antagonists to block the expression of host antiviral proteins. In addition, CoV nsp14 and nsp16 exhibit N7-methyltransferase (N7-MTase) and 2’O-methyltransferase activities, respectively, which catalyze the formation of a 5’cap-1 structure, preventing recognition of viral RNA by PRRs (Chen et al. 2009; Decroly et al. 2008). All CoV nsp14s have 3′-to-5′ exoribonuclease (ExoN) activity and N7-methyltransferase activity (N7-MTase) (Chen et al. 2009; Minskaia et al. 2006). N7-MTase activity is critical for translation of the viral genome and prevents the sense of viral mRNAs as a “nonself” signature by host PRRs (Becares et al. 2016). ExoN activity is critical for the fidelity of viral replication (Minskaia et al. 2006). Previous studies have suggested that CoV nsp14 plaied potential roles in modulation of innate immunity (Becares et al. 2016; Case et al. 2017). Mutation of N7-MTase domain of murine hepatitis virus (MHV) nsp14 enhances its sensitivity to the host innate immune response, and ExoN activity of nsp14 is essential for its resistance to the antiviral innate immune response (Case et al. 2017). A recent study showed that N-7 MTase-deficient PEDV was defective in replication, but infection with this virus resulted in increased secretion of type I and III IFNs (Lu et al. 2020). However, the role and regulatory mechanisms of PEDV nsp14 in innate immunity are still poorly understood.

PEDV is an alphacoronavirus that causes acute and highly contagious enteric viral disease in pigs. Starting in 2010, a significant increase in PEDV outbreaks occurred in the USA and Asia countries, giving rise to severe economic consequences worldwide (Sun et al. 2012; Tian et al. 2014). Variants of highly virulent PEDV strains that have contributed to the occurrence of these outbreaks were identified. The immune evasion of these PEDV strains is closely associated with severe clinical pathogenesis. Several PEDV proteins have been reported to interrupt RIG-I signaling pathway to inhibit IFN-β production.

Although RIG-I signaling pathways regulated by PEDV viral proteins have been extensively characterized, how PEDV regulates proinflammatory cytokine expression remains largely unknown. Here, in this study, we identified PEDV nsp14 as an NF-κB antagonist. This research showed that nsp14 suppressed NF-κB signaling by interacting with IKKα/β and p65 as well as interfering with the phosphorylation of IKKα/β and IκBα, which subsequently blocked the degradation of IκBα and thus suppressed TNF-α-induced p65 phosphorylation and nuclear translocation. Overall, our data identified a new NF-κB pathway antagonist of PEDV and a novel antagonistic mechanism evolved by PEDV to inhibit proinflammatory cytokine expression.

## Results

### PEDV nsp14 inhibits SeV infection-induced NF-κB promoter activation

**Fig. 1:**
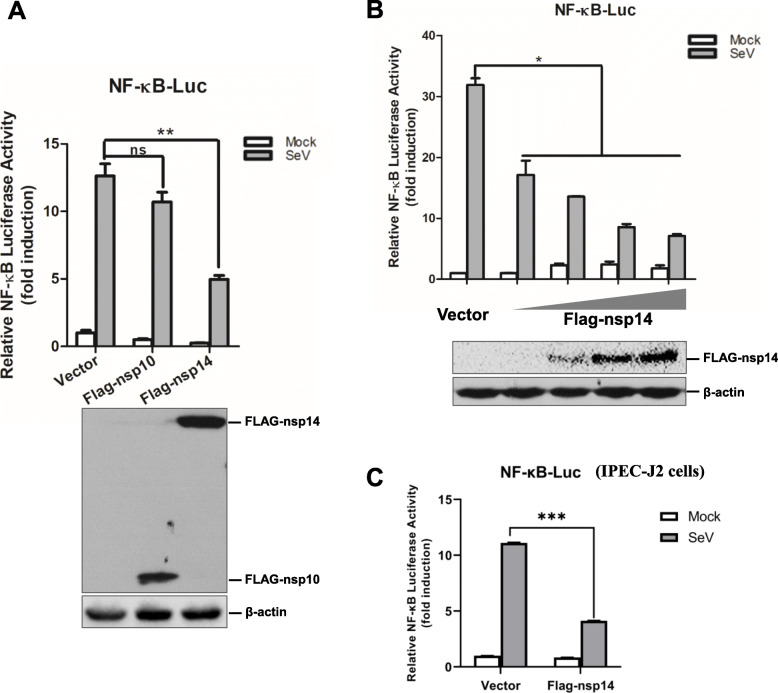
PEDV nsp14 inhibites NF-κB promoter activation. **A** HEK-293 T cells were transfected with pNF-κB-Luc reporter plasmid (0.05 μg) and pRL-TK plasmid (0.005 μg) together with empty vector plasmids or plasmids expressing PEDV nsp10 or nsp14 (0.1 μg) for 24 h. Cells were then mock-infected or infected with SeV for another 16 h, and luciferase activities were measured. Expression of nsp10 and nsp14 was analyzed by Western blotting using an anti-Flag antibody. **B** HEK-293 T cells were transiently transfected with pNF-κB-Luc reporter plasmid (0.05 μg) and pRL-TK plasmid (0.005 μg) together with increasing amounts of plasmids expressing PEDV nsp14 (0, 0.01, 0.05, 0.1 or 0.2 μg). At 24 hpt, cells were mock-infected or infected with SeV for another 16 h. Luciferase activities were measured using a dual luciferase reporter assay. Expression of nsp14 was analyzed via Western blotting with an anti-Flag antibody. Data are expressed as the means ± SD from three independent experiments. *, *P < 0.05*; **, *P < 0.01*. ns indicates not significant. **C** IPEC-J2 cells were transiently transfected with pNF-κB-Luc reporter plasmid and pRL-TK plasmid together with empty vector plasmids or plasmids expressing PEDV nsp14 for 24 h. Cells were then mock-infected or infected with SeV for another 24 h, and luciferase activities were measured

PEDV has the ability to modulate host immune and inflammatory responses (Cao et al. 2015; Zhang et al. 2017a, 2017b). PEDV infection significantly inhibits NF-κB activation and the production of proinflammatory cytokines in porcine epithelial cells (Zhang et al. 2017a, 2017b). nsp14 is a potential viral factor that blocks NF-κB activation; however, the mechanism remains unknown (Zhang et al. 2017a, 2017b). To evaluate the effect of PEDV nsp14 on NF-κB activation, a dual luciferase reporter assay was performed to determine the antagonistic effect of nsp14 on NF-κB promoter activation induced by Sendai virus (SeV). PEDV nsp10 that has no effect on NF-κB activation (Zhang et al. 2017a, 2017b), was used as a control. Expression of PEDV nsp10 and nsp14 was confirmed by Western blotting (Fig. 1A). The activation of NF-κB promoter induced by SeV was significantly inhibited by PEDV nsp14 but not by nsp10 (Fig. 1A). In addition, inhibitory effect of nsp14 on NF-κB activation was dose dependent (Fig. 1B). Overexpression nsp14 suppressed activation of NF-κB promoter in porcine intestinal epithelial cells (IPEC-J2 cell line) (Fig. 1C). These data suggest that nsp14 is an important antagonistic factor of PEDV to inhibit NF-κB activation.

### PEDV nsp14 blocks the expression of NF-κB-mediated proinflammatory cytokines

**Fig. 2:**
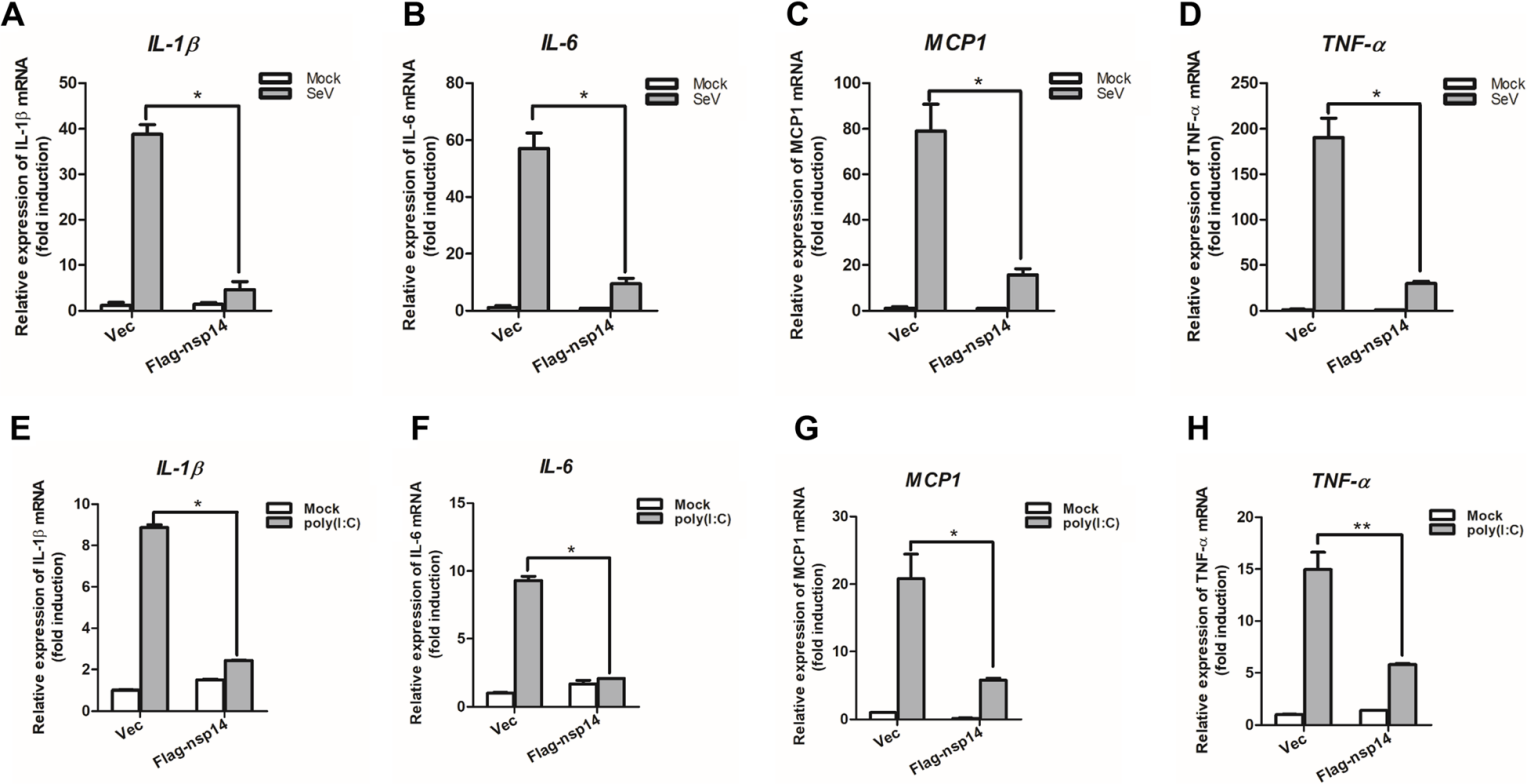
PEDV nsp14 suppressed the expression of proinflammatory cytokines in HEK293T cells. **A-D** HEK293T cells were transfected with 1 μg vector plasmids or Flag-nsp14 expression plasmids. At 24 hpt, cells were infected with SeV for 16 h, andmRNA levels of IL-1β (**A**), IL-6 (**B**), MCP1 (**C**) and TNF-α (**D**) were measured *via* qPCR analysis. **E-F** HEK293T cells were transfected with 1 μg vector plasmids or Flag-nsp14 expression plasmids. At 24 hpt, cells were transfected with 1 μg poly (I:C) for 12 h, and mRNA expression levels of IL-1β (**E**), IL-6 (**F**), MCP1 (**G**), and TNF-α (**H**) were measured *via* qPCR analysis. *, *P < 0.05*; **, *P < 0.01*

**Fig. 3:**
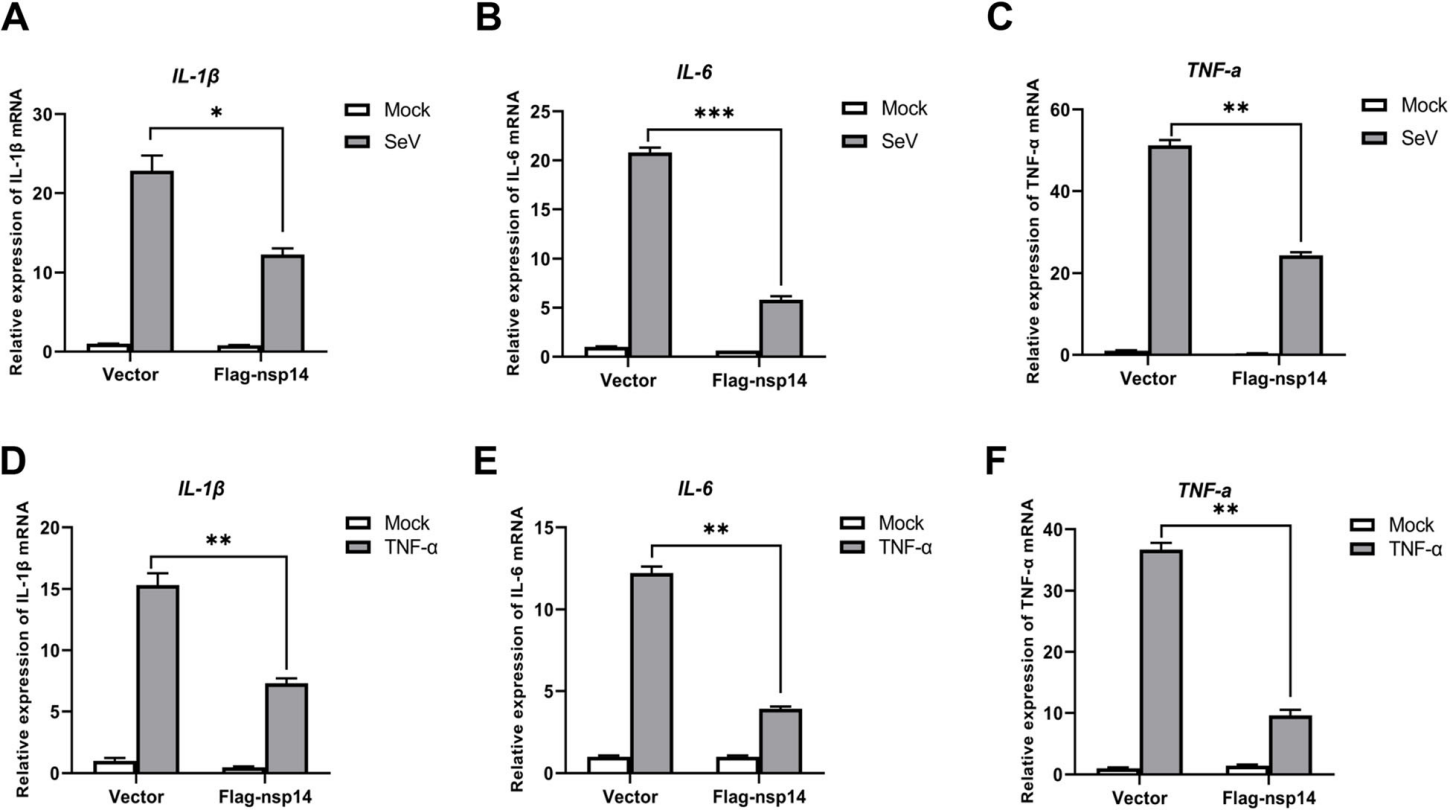
PEDV nsp14 suppressed the expression of proinflammatory cytokines in porcine intestinal epithelial cells. **A-C** IPEC-J2 cells were transfected with 1 μg vector plasmids or Flag-nsp14 expression plasmids. At 24 hpt, cells were infected with SeV for 24 h, and mRNA levels of IL-1β (**A**), IL-6 (**B**) and TNF-α (**C**) were measured *via* qPCR analysis. **D**, **E** IPEC-J2 cells were transfected with 1 μg vector plasmids or Flag-nsp14 expression plasmids. At 24 hpt, cells were treated with TNF-α (30 ng/mL) or mock-treated for 8 h, and mRNA expression levels of IL-1β (**D**), IL-6 (**E**) and TNF-α (**F**) were measured *via* qPCR analysis. *, *P < 0.05*; **, P < 0.01; *** P < 0.001

As described above, PEDV nsp14 suppressed activation of NF-κB promoter. To determine if nsp14 inhibits the expression of proinflammatory cytokines, HEK-293 T cells were transfected with p3xFLAG-CMV 7.1 vector plasmids or Flag-nsp14 expression plasmids for 24 h and then mock-infected or infected with SeV for 16 h. SeV infection strongly induced the expression of IL-1β, IL-6, MCP1 and TNF-α (Fig. 2A-D). However, expression of these proinflammatory cytokines was significantly inhibited in the presence of PEDV nsp14. These data indicate that PEDV nsp14 decreases the expression of pro-inflammatory cytokines induced by NF-κB. To further confirm the nsp14-mediated inhibitory effect on proinflammatory cytokine expression, HEK-293 T cells were transfected with poly (I:C) to activate NF-κB signaling pathway, and the expression of proinflammatory cytokines in the presence or absence of PEDV nsp14 was evaluated *via* qPCR. Poly (I:C) transfection remarkably induced the expression of IL-1β, IL-6, MCP1 and TNF-α. However, expression of these proinflammatory cytokines was considerably suppressed by PEDV nsp14 (Fig. 2E-H). The regulatory effect of nsp14 on mRNA expression of IL-1β, IL-6 and TNF-α induced by SeV and TNF-α in IPEC-J2 cells was further evaluated, and the results showed that overexpression of nsp14 considerably inhibited the expression of these proinflammatory cytokines induced by both SeV (Fig. 3A-C) and TNF-α (Fig. 3D-F) in porcine intestinal epithelial cells. These data confirm that PEDV nsp14 blocks the expression of pro-inflammatory cytokines mediated by NF-κB.

### PEDV nsp14 does not downregulate the protein levels of various

#### NF-κB signaling pathway components

**Fig. 4:**
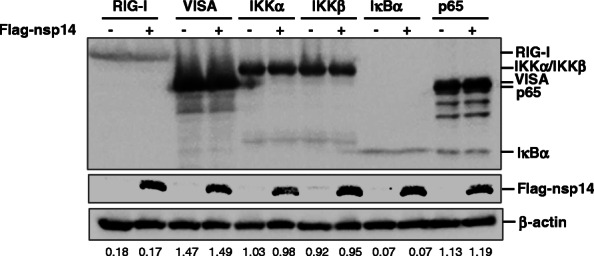
PEDV nsp14 did not inhibit protein expression of various components of NF-κB pathway. HEK293T cells were transfected with constructs expressing HA-tagged RIG-I, VISA, IKKα, IKKβ, IκBα or p65 along with vector plasmids or Flag-nsp10 expression plasmids. At 24 hpt, cell lysates were prepared for Western blotting analysis with the indicated antibodies. The relative abundance of the detected proteins was determined by densitometric analysis using ImageJ software

Activation of RIG-I-mediated NF-κB pathway requires many components, including RIG-I, VISA, IKKα, IKKβ, IκBα and p65. Protein abundance of these signaling components directly affects activation of NF-κB pathway. To investigate the component that may potentially be targeted by PEDV nsp14, effect of nsp14 on the expression of RIG-I, VISA, IKKα, IKKβ, IκBα and p65 were investigated. HEK-293 T cells were cotransfected with plasmids expressing Flag-nsp14 or vector plasmids along with plasmids encoding HA-tagged adaptor molecules (RIG-I, VISA, IKKα, IKKβ, IκBα or p65). Protein abundance of these molecules was evaluated *via* Western blotting analysis. As in Fig. 4, PEDV nsp14 did not affect protein expression of various components of NF-κB signaling pathway.

#### PEDV nsp14 interactes with IKKα, IKKβ and p65

**Fig. 5:**
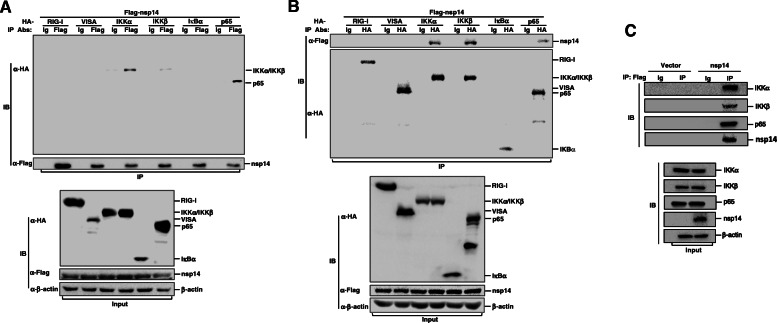
PEDV nsp14 interactes with IKKα, IKKβ and p65 in NF-κB pathway. **A** and **B** HEK-293 T cells were cotransfected with Flag-nsp14 expression plasmids and the indicated HA-tagged molecule expression plasmids (RIG-I, VISA, IKKα, IKKβ, IκBα or p65). At 36 hpt, cell lysates were prepared for Co-IP experiments using anti-Flag antibody (**A**), anti-HA antibody or (**B**) nonspecific mouse IgG. Western blotting analysis was carried out with the indicated antibodies to evaluate the whole cell lysates (WCLs) and immunoprecipitation complexes. **C** IPEC-J2 cells were transfected with Flag-nsp14 expression plasmids for 36 h. Then, cell lysates were prepared for Co-IP experiments using anti-Flag antibody or nonspecific mouse IgG. Western blotting analysis was carried out with the indicated antibodies to evaluate WCLs and IP complexes

PEDV nsp14 does not affect protein expression of the components of NF-κB signaling pathway. We further evaluated whether nsp14 interacts with any of these components. The interaction status between PEDV nsp14 and these components was investigated using Co-IP assays. HEK-293 T cells were transfected with vector plasmids or Flag-nsp14 expression plasmids and with plasmids expressing HA-tagged RIG-I, VISA, IKKα, IKKβ, IκBα or p65. The lysates were pulled down with anti-Flag or IgG antibody and subjected to Western blotting analysis using the indicated antibodies. As shown in Fig. 5A, IKKα, IKKβ and p65 were pulled down by nsp14. A reverse Co-IP experiment was further performed to confirm the interaction. Similarly, nsp14 was efficiently pulled down by IKKα, IKKβ and p65 (Fig. 5B). Interaction between nsp14 and IKKα, IKKβ or p65 was also evaluated in porcine IPEC-J2 cells and got the same conclusion (Fig. 5C). These data indicate that nsp14 interacts with IKKα, IKKβ and p65.

#### PEDV nsp14 inhibites IKKα/β phosphorylation and decreased NF-κB nuclear translocation

**Fig. 6:**
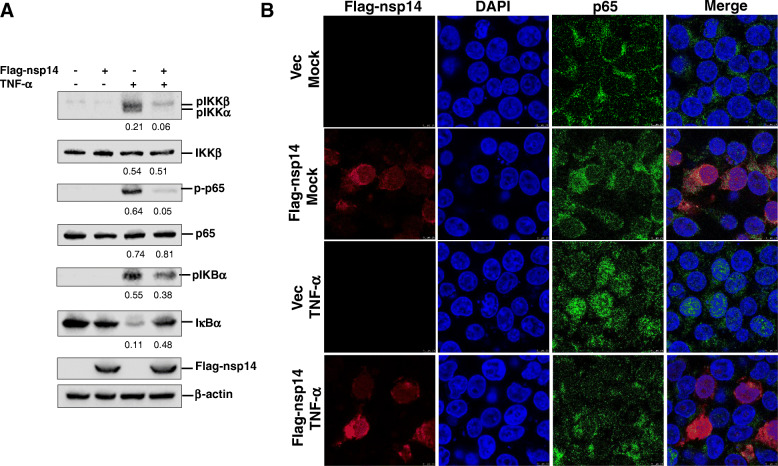
PEDV nsp14 inhibites IKKα/β and p65 phosphorylation and nuclear translocation of NF-κB. **A** HEK293T cells were transfected with 1 μg vector plasmids or Flag-nsp14 expression plasmids. At 24 hpt, cells were treated with TNF-α (30 ng/mL) or mock-treated for 30 min. Cell lysates were then prepared, and the target proteins were detected by Western blotting. **B** Double immunostained of transfected HEK293T cells treated in the same way as with anti-Flag (red) and anti-p65 (green) antibodies

TNF-α is an effective agonist of NF-κB signaling pathway. TNF-α treatment results in activation of downstream IKKs (Li et al. 2009). IκBα is phosphorylated by the activated IKKs, resulting in IκBα ubiquitination and degradation, and then, p65/p50 dimer is released from IκBα and translocated into nucleus (Chan et al. 2000; Pires et al. 2018; Rahman and McFadden 2011). To gain insights into the molecular mechanisms by which nsp14 suppresses NF-κB activation, effect of nsp14 on IKKα/β, IκBα and p65 phosphorylation was examined. HEK-293 T cells were transfected with vector plasmids or Flag-nsp14 expression plasmids. At 24 h post transfection (hpt), cells were treated with TNF-α for 30 min, and then, phosphorylation levels of IKKα/β, IκBα and p65 were assessed *via* Western blotting. As shown in Fig. 6A, TNF-α stimulation led to a significant increase in IKKα/β, IκBα and p65 phosphorylation, but overexpression PEDV nsp14 remarkably inhibited this process. Meanwhile, TNF-α treatment downregulated the expression of IκBα, while nsp14 restored IκBα expression in TNF-α-stimulated cells (Fig. 6A). These results indicate that nsp14 restrains phosphorylation of IKK complex, which in turn blocks NF-κB activation.

Nuclear translocation of p65 is an indicator of NF-κB activation. Subsequently, nuclear translocation of p65 in response to TNF-α treatment in absence and presence of nsp14 were assessed using an indirect immunofluorescence assay. TNF-α treatment caused increased nuclear translocation of p65. However, overexpression PEDV nsp14 decreased TNF-α-induced nuclear translocation of p65 (Fig. 6B). Meanwhile, colocalization between nsp14 and p65 was also observed. These results suggest that PEDV nsp14 inhibits NF-κB nuclear translocation and contributes to the suppression of NF-κB pathway activation.

#### Overexpression nsp14 protein enhances PEDV replication

**Fig. 7:**
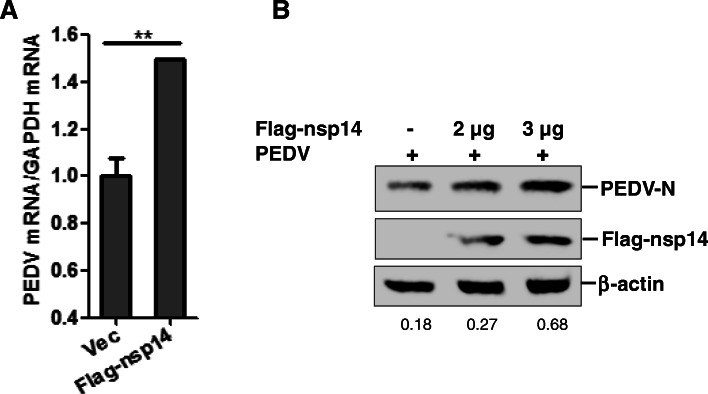
PEDV nsp14 promotes PEDV replication. **A** PK-15 cells were transfected with 2 μg vector plasmids or Flag-nsp14 expression plasmids. At 24 hpt, cells were infected with PEDV at an MOI of 1 for 18 h. The cells were then collected and subjected to qPCR analysis. **B** Western blotting analysis of transfected PK-15 cells using the indicated antibodies. The relative abundance of PEDV N protein was determined by densitometric analysis using ImageJ software

As a vital nonstructural protein, PEDV nsp14 plays important roles during viral infection. The results above showed that nsp14 suppressed NF-κB activaton and decreased early production of proinflammatory cytokines. To evaluate PEDV replication activity in cells overexpressing nsp14-, PEDV replication status in PK-15 cells was investigated after transfection with nsp14 expression plasmids. Cells were incubated with PEDV at an MOI of 1 at 24 hpt and infected for 18 h. Viral mRNA (Fig. 7A) and viral protein levels (Fig. 7B) were determined and used as an indicator of viral replication. Overexpression nsp14 considerably enhanced PEDV replication. Together, these data suggest that nsp14 plays an important role in PEDV replication.

## Discussion

The most important function of NF-κB in the biological system is regulation of immune responses. It plays pivotal roles in immune homeostasis by regulating innate immune responses, inflammation, cell survival and cell proliferation (Poppe et al, 2017; Wullaert et al. 2011a, 2011b). Canonical NF-κB pathway is mediated mainly by PRRs and proinflammatory cytokine receptors, which induce degradation of the inhibitory factor IκBα. The release of IκBα from NF-κB complex contributes to nuclear translocation of NF-κB, thus activates the expression of proinflammatory cytokines (Khatiwada et al. 2017; Rothwarf et al. 1998). Recognition of PAMPs by PRRs triggers host innate immune responses through activation of signaling cascades which eventually induce inflammatory responses and eradicate pathogens (Kumar et al. 2011; O'Neill and Bowie 2010). Hence, to counteract the host antiviral response, many viruses have evolved various strategies to evade NF-κB signaling (Smits et al, 2010).

Virion-associated protein 119 of virus inhibits NF-κB activation by interacting with NEMO, which finally results in a decrease in p65 nuclear translocation (Nagendraprabhu et al. 2017). Herpes simplex virus UL24, UL36, UL42, US3 and ICP0 proteins inhibit NF-κB activation by blocking the nuclear translocation of NF-κB or preventing the degradation of IκBα (Wang et al. 2014; Xu et al. 2017; Ye et al. 2017; Zhang et al. 2013a; Zhang et al. 2013b). MERS-CoV 4b protein interferes with NF-κB-mediated innate immune response by binding karyopherin-α4 (KPNA4), thus preventing NF-κB nuclear translocation (Canton et al. 2018). In this study, we demonstrates that PEDV nsp14 interacts with IKKα/β and p65 to inhibit NF-κB activation by inhibiting IKKα/β phosphorylation and decreasing NF-κB nuclear translocation, thereby downregulating the transcription of various proinflammatory cytokines. As expected, overexpression PEDV nsp14 promoted viral replication. Thus, we deduced that PEDV nsp14-mediated suppressive effect on NF-κB activity is associated with PEDV-induced pathology and virulence *in vivo*.

All CoVs encode a bifunctional nsp14 protein containing 3′-to-5′ exoribonuclease and N7-methyltransferase activities, which are essential for viral replication fidelity, mRNA stability, and translation (Chen et al. 2009; Minskaia et al. 2006). Function of SARS-CoV nsp14 has been widely studied (Chen et al. 2009; Chen et al. 2013; Jin et al. 2013; Ma et al. 2015). SARS-CoV is a *betacoronavirus* and thus differs from *alphacoronavirus*. A study on TGEV, a porcine *alphacoronavirus*, revealed that a recombinant virus with a mutation in the TGEV ExoN domain induced a reduced antiviral response compared to its parental virus (Becares et al. 2016). This suggested that porcine CoV nsp14 ExoN activity may be involved in regulation of innate immunity. In contrast, ExoN and N-7 MTase activity of *betacoronavirus* MHV play roles in suppression of the innate immune response (Case et al. 2016; Case et al. 2017). However, function of nsp14 proteins of other *alphacoronaviruses* remains largely unknown. A recent study characterized N-7 MTase of PEDV nsp14, which suggested that it is attenuated and triggers stronger production of type I and III IFNs (Lu et al. 2020). These findings indicated that PEDV nsp14 N-7 MTase may be involved in the suppression of host innate immunity. Our data demonstrate that PEDV nsp14 inhibits NF-κB activity by targeting IKK complex and p65. nsp4 might block the formation of NF-κB complex and then inhibit the expression of various proinflammatory cytokines. These results support the hypothesis that PEDV nsp14 could be a target for the development of live attenuated vaccine candidates and antiviral therapeutics.

Relationship between PEDV infection and innate immunity has been reported previously. PEDV infection inhibits type I IFN production and early production of proinflammatory cytokines (Zhang et al. 2017a, 2017b; Zhang et al. 2016). PEDV N protein, nsp1, PLP2 and nsp5 are responsible for the suppression of type I IFN production *via* different mechanisms (Wang et al. 2016; Zhang et al., 2018; Zhang et al. 2017a, 2017b; Zhang et al. 2016; Zheng et al. 2008). PEDV nsp1 suppresses the phosphorylation of IκBα and blocks IκBα degradation, which in turn inhibits p65 activation, but PEDV nsp1 does not affect phosphorylation of IKKα/β complex or interacts with IKKα/β (Zhang et al. 2017a, 2017b). Here, we report that PEDV nsp14 interacts with IKKα/β and p65 to inhibit phosphorylation of IKKα/β complex, which in turn blocks nuclear translocation of NF-κB. This result suggests that both PEDV nsp14 and nsp1 regulate NF-κB signaling *via* different mechanisms.

## Conclusions

**Fig. 8:**
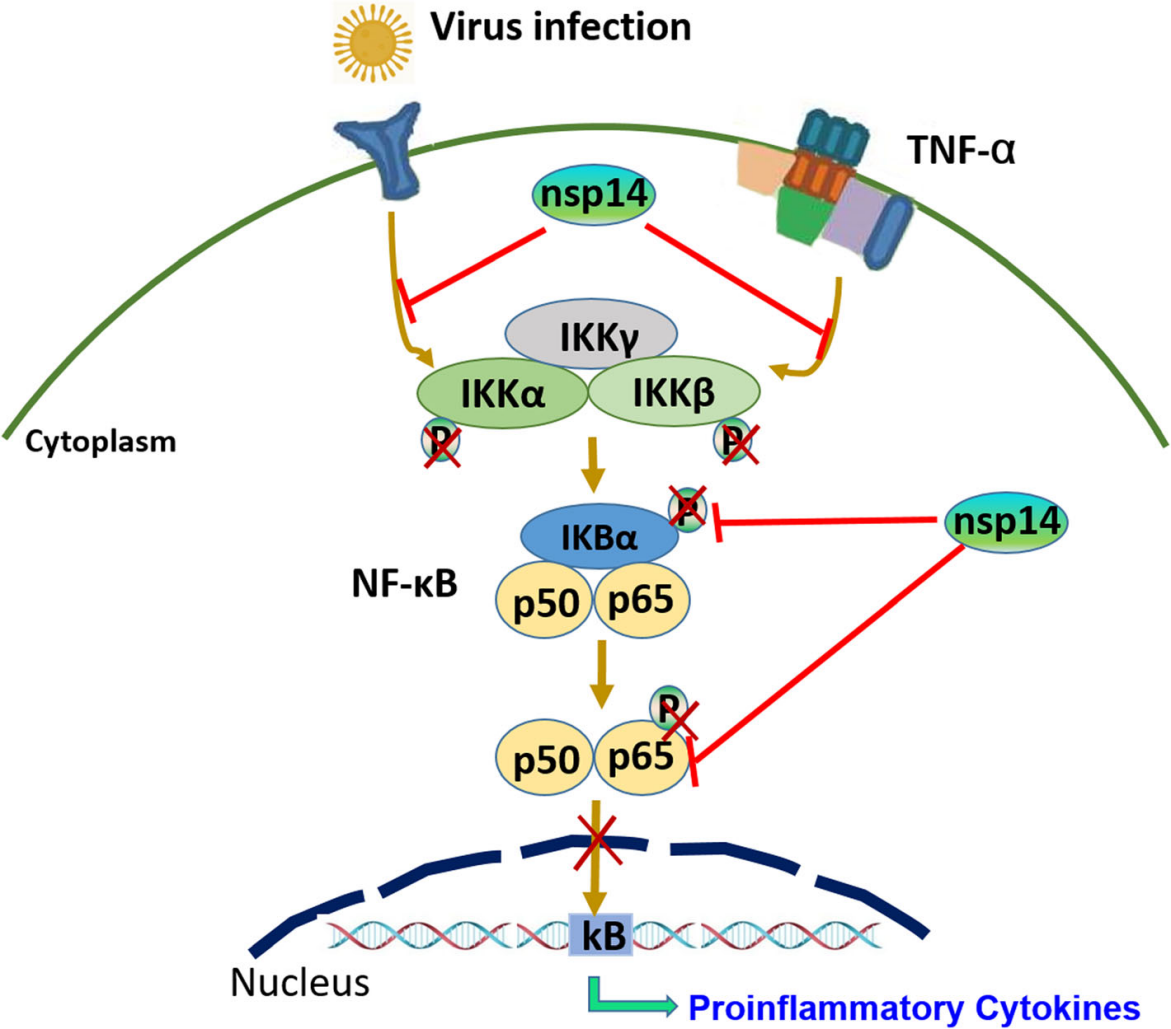
Schematic diagram of PEDV nsp14 targets IKK complex and p65 to inhibit NF-κB activity. PEDV nsp14 negatively regulates NF-κB signaling pathway by interacting with IKKα/β and p65, which inhibits the phosphorylation of IKKα/β and p65, and nuclear translocation of NF-κB, leading to the decreased expression of early production of proinflammatory cytokines

In conclusion, this work identified PEDV nsp14 as a new NF-κB antagonist Nsp14 suppresses NF-κB activity and decreases early production of proinflammatory cytokines by interacting with IKKα/β and p65, which inhibits the phosphorylation of IKKα/β and p65 and nuclear translocation of NF-κB (Fig. 8).

## Methods

### Cells

HEK-293 T, PK-15 and IPEC-J2 cells were maintained at 37 °C with 5% CO_2_ in Dulbecco’s modified Eagle’s medium (DMEM, Gibco) supplemented with 10% heat-inactivated fetal bovine serum (ExCell, FSP500).

### Plasmid constructs

cDNA fragments encoding PEDV nsp10 and nsp14 were amplified from PEDV and inserted into a p3xFLAG-CMV 7.1 eukaryotic expression vector to generate Flag-tagged nsp10- and nsp14 expression plasmids. All constructed plasmids were confirmed by DNA sequencing.

### RNA extraction and RT–qPCR

Monolayer HEK-293 T cells seeded in 12-well plates were transfected with vector plasmids or Flag-nsp14 expression plasmids. At 24  hpt, cells were mock-infected or infected with SeV for 16 h and then washed with PBS to remove cell debris. Total RNA was prepared and used for cDNA synthesis as described previously (Zhang et al. 2017a, 2017b). cDNA was then employed for quantitative PCR (qPCR) using ChamQ™ Universal SYBR® qPCR reagents (Vazyme) and an ABI QuantStudio5 real-time PCR system. The qPCR primers are listed in Table 1. Data were normalized to GAPDH expression. The relative expression of mRNA was evaluated using the 2^−∆∆Ct^ threshold method (Livak and Schmittgen 2001).

**Table 1:** qPCR primers used in the present study

Primers	Sequences
human-IL-β-F	5′- CAAAGGCGGCCAGGATATAA-3´
human-IL-β-R	5′- CTAGGGATTGAGTCCACATTCAG-3´
human-IL-6-F	5′- TGACCCAACCACAAATGC-3´
human-IL-6-R	5′- AGGAACTCCTTAAAGCTGCG-3´
human-MCP1-F	5′- AGGTGACTGGGGCATTGATTG-3´
human-MCP1-R	5′- GTCTCTGCCGCCCTTCTGTG-3´
human-TNF-F	5′- AGAGGGAGAGAAGCAACTACA-3´
human-TNF-R	5′- GGGTCAGTATGTGAGAGGAAGA-3´

### Antibodies and commercial cytokine

Antibodies used for Western blotting analysis and immunofluorescence assays (IFAs) included anti-HA mouse antibody (Invitrogen), anti-Flag mouse antibody (Invitrogen), anti-human IKKβ rabbit antibody, anti-pIKKα/IKKβ rabbit antibody, anti-NF-κB rabbit antibody, anti-pNF-κB rabbit antibody, anti-IκBα mouse antibody, and anti-pIκBα rabbit antibody (Cell Signaling Technology). TNF-α (InvivoGen) was used to activate NF-κB pathway at a final concentration of 30 ng/mL.

### Western blotting

HEK-293 T cells were cotransfected with vector plasmids or Flag-nsp14 along with HA-tagged adaptor molecule expression plasmids (RIG-I, VISA, IKKα, IKKβ, IκBα and p65) using Lipofectamine 2000. At 24 hpt, cells were lysed with NP-40 lysis buffer supplemented with complete protease inhibitors (Roche), and then, cell lysates were boiled at 95 °C for 5 min. The supernatant was then obtained by centrifugation. For detection of phosphorylated proteins, the transfected cells were treated with TNF-α for 30 min and lysed in the presence of an additional phosphatase inhibitor (Thermo Scientific Pierce). All samples were separated, transferred to nitrocellulose membranes (Millipore) and then incubated with the appropriate primary antibodies overnight at 4 °C after blocking. Appropriate HRP-conjugated secondary antibodies (Santa Cruz Biotechnology) were used to generate antigen-antibody complexes, which were visualized using ECL reagents (Advansta).

### Coimmunoprecipitation assay

HEK-293 T cells were cotransfected with vector or Flag-nsp14 along with HA-tagged adaptor molecule expression plasmids (RIG-I, VISA, IKKα, IKKβ, IκBα and p65) using Lipofectamine 2000 for 36 h. The cells were then lysed in lysis buffer supplemented with complete protease inhibitors (Roche). IPEC-J2 cells were transfected with vector or Flag-nsp14 using Lipofectamine 2000 for 36 h. The cells were then lysed in lysis buffer supplemented with complete protease inhibitors (Roche). Cell lysates were incubated with 50 μL protein G agarose beads and appropriate antibodies overnight at 4 °C. After three washes of agarose beads, the immunoprecipitated complexes were subjected to Western blotting analysis.

### Immunofluorescence assay

HEK-293 T cells were transfected with vector plasmids or Flag-nsp14 expression plasmids. At 24 hpt, cells were stimulated with TNF-α for 30 min and fixed with 4% paraformaldehyde. Cells were then permeabilized and incubated with appropriate antibodies as previously described (Nagendraprabhu et al. 2017). The nuclei were stained with DAPI (4′6´-diamidino-2-phenylindole) (Sigma) for 10 min. The stained cells were analyzed using a Leica TCS SP5 II AOBS confocal microscope at the appropriate settings.

### Luciferase reporter assay

HEK-293 T cells were cotransfected with HA- or Flag-tagged protein expression plasmids, together with pNF-κB luciferase reporter plasmid and Renilla internal control plasmids. IPEC-J2 cells were cotransfected with Flag vector or Flag-nsp14 and pNF-κB-Luc reporter plasmids as well as pRL-TK plasmids. The cell lysates were collected to measure the luciferase activity using a Dual Luciferase Assay System Kit (Promega) according to the manufacturer’s instructions. The data indicate the relative firefly luciferase activity value normalized to the Renilla luciferase activity value.

### Statistical analysis

Standard two-tailed unpaired Student’s *t* tests were employed to determine the significance of differences between groups. All results are presented as the means ± standard deviation. A *P*-value less than 0.05 was considered statistically significant.

## Data Availability

Data will be shared upon request by the readers.
